# Differential gene expression in mouse retina related to regional differences in vulnerability to hyperoxia

**Published:** 2010-04-28

**Authors:** Yuan Zhu, Riccardo Natoli, Krisztina Valter, Jonathan Stone

**Affiliations:** 1 ARC Centre of Excellence in Vision Science, Canberra, Australia; 2 Division of Biomedical Sciences and Biochemistry, Research School of Biology, The Australian National University, Canberra, Australia; 3 Save Sight Institute, Bosch Institute and Discipline of Physiology, The University of Sydney, Sydney, Australia

## Abstract

**Purpose:**

In the C57BL/6J mouse retina, hyperoxia-induced degeneration of photoreceptors shows strong regional variation, beginning at a locus ~0.5 mm inferior to the optic disc. To identify gene expression differences that might underlie this variability in vulnerability, we have used microarray techniques to describe regional (superior-inferior) variations in gene expression in the retina.

**Methods:**

Young adult C57BL/6J mice raised in dim cyclic illumination (12 h at 5 lx and 12 h in darkness) were exposed to hyperoxia (75% oxygen for two weeks). Retinas were collected from hyperoxia-exposed and control animals without fixation and divided into superior and inferior halves. RNA was extracted from each sample, purified, and hybridized to Mouse Gene 1.0 ST arrays (Affymetrix). The consistency of the microarray results was assessed using quantitative PCR for selected genes. Expression data were analyzed to identify genes and ncRNAs whose differential expression between the superior and inferior retina could be associated with relative vulnerability to hyperoxia.

**Results:**

In control retinas, only two genes showed a fold difference in expression >2 between the superior and inferior retina; another 25 showed a fold difference of 1.5–2.0. Of these 27, the functions of six genes, including ventral anterior homeobox containing gene 2 (*Vax2)* and T-box 5 (*Tbox5),* are related to parameters of anatomic development and the functions of five are related to sensory perception. Among the latter, short-wave-sensitive cone opsin (*Opn1sw*) was more strongly expressed in the inferior retina and medium-wave-sensitive cone opsin (*Opn1mw*) in the superior retina. This is consistent with known differences in S- and M-cone distribution, confirming our separation of retinal regions. The highest fold difference was reported for membrane metalloendopeptidase (*Mme)*, a member from the metallothionein group of cytoprotective proteins. To identify genes whose regulation by hyperoxia was significantly different between the inferior and superior retina, we calculated the “fold margin” (FM, the difference between hyperoxia-induced regulation in the inferior and superior retina) for each gene, and identified genes for which abs(FM) > 0.5. Genes thus identified numbered 112, and included many immune-, cell defense-, and inflammation- related genes.

**Conclusions:**

Gene expression analysis revealed relatively subtle differences between inferior and superior regions of control C57BL/6J retinas, with only 27 genes showing an expression difference >1.5 fold. Among these, genes related to cytoprotection and apoptosis were included, along with genes related to central projections and cone-type differences. After hyperoxia-induced photoreceptor degeneration had begun, the number of genes that showed significant expression differences between the inferior and superior retina more than quadrupled, with genes related to immune processes, defense processes, and inflammation being numerically dominant.

## Introduction

Tissue oxygen levels play an important role in the development of the retina and in the stability of photoreceptors. During development, the retina is initially avascular and is supplied with oxygen by diffusion from choroidal circulation. As photoreceptors develop and commence functioning in rodents during the second week of postnatal life [1], their consumption of oxygen increases steeply and their retinas become hypoxic. The onset of hypoxia is the stimulus for vasculogenesis [2], mediated by the potent hypoxia-induced angiogenic vascular endothelial growth factor (VEGF) [3]. The formation of retinal vessels relieves the hypoxia induced by photoreceptor activity. During this period, oxygen regulates a naturally occurring process of photoreceptor death in a linear way, hypoxia accelerating and hyperoxia decelerating the rate at which photoreceptors die [4]. As retinas mature, however, hyperoxia becomes toxic; both hypoxia and hyperoxia destabilize photoreceptors [5]. The toxic impact of hyperoxia on photoreceptors was first reported in adult rabbits [6] and subsequently in rodents [7,8]. Hyperoxia also induces the death of endothelial cells and the thinning of retinal vasculature and reduces VEGF expression (thus the opposite of the angiogenic effect of hypoxia) [9].

There is also evidence that hyperoxia can improve the stability of photoreceptors. In experimental retinal detachment, hypoxia caused by the separation of the outer retina from its normal source of nutrients is a factor in inducing the death and deconstruction of photoreceptors, as well as in reducing glial proliferation. Hyperoxia mitigates all these pathologies [10,11]. Hyperbaric oxygen therapy (HBO), which involves very high tissue oxygen levels for short periods, has been reported to have long-lasting benefits for patients with retinitis pigmentosa [12] and several other ophthalmic diseases, including glaucoma, macular detachment, and neovascular disease [13]. In recent work undertaken to resolve this apparent discrepancy [8,14], we have noted that the impact of hyperoxia on retinal gene expression is biphasic, initially regulating protective genes and later regulating genes related to photoreceptor death. This suggests that hyperbaric oxygen protects the retina because it is produced briefly, typically lasting only a few hours a day, and may regulate only the protective cohort of genes.

This study probes the mechanism of oxygen-induced destabilization of photoreceptors by exploiting known regional differences in photoreceptor vulnerability. A superior-inferior gradient of vulnerability is evident for hyperoxia, which in C57BL/6J mice affects photoreceptors in the inferior retina more severely [14,15]. Other works have described a periphero-central gradient in photoreceptor vulnerability to hyperoxia, both hyperbaric [16] and normobaric [7]. It is possible that these researchers noted the central location of vulnerable photoreceptors, without noting whether they were located in the superior or inferior retina.

Several gradients of vulnerability of the retina have been previously reported. For example, light-induced retinal damage can be more severe in the superior retina than the inferior retina of albino rats [17,18]. Degeneration induced by the photoreceptor toxin iodoacetic acid is more severe in the inferior retina and visual streak of rabbit retinas [19] and, in a genetically degenerative mouse model (the *rd/rd* mouse), significantly more cones survive in the inferior than superior hemisphere in most retinas [20]. Although various factors, such as light history, rhodopsin content, and fatty acid composition have been reported to influence the vulnerability of photoreceptors to damage, the mechanisms responsible for regional variations of photoreceptor vulnerability to hyperoxia remain unknown.

## Methods

### Animals and hyperoxic damage

C57BL/6J mice, a hyperoxia-vulnerable strain [21], were used for this study. Mice were raised in dim cyclic illumination (12 h at 5 lx and 12 h in dark) and maintained in this level of lighting for the duration of the experiment. At the postnatal age (P) of P83–P90 days, some mice were exposed to constant hyperoxia (75% O_2_) for 14 days. During exposure, the mice were given free access to food and water and were housed in plexiglass chambers, in which the oxygen level was controlled by a feedback device (OxyCycler; Reming Bioinstruments, Redfield, NY).

### RNA isolation and purification

Oxygen-exposed animals were euthanized at the end of the period of hyperoxia; controls were euthanized at the same age. Retinas were removed and divided into superior and inferior halves. Retinal samples from two animals (one male and one female) were pooled for each condition (superior and inferior retina from the controls, superior and inferior retina exposed). RNA extraction was performed using TRIzol Reagent (Invitrogen™ Life Technologies, Carlsbad, CA) and an RNAqueous-Micro Kit (Ambion, Foster City, CA). TRIzol was used to isolate the RNA and the RNAqueous-Micro Kit was used to purify and DNase-treat the RNA. Retinas were quickly placed into a 1.5 ml tube containing 200 µl of TRIzol. Following the homogenization of retinas on ice, a further 660 µl of TRIzol and 160 µl of chloroform were added to the tube. The tube was vortexed for 20 s and allowed to stand for 7 min at room temperature. The tubes were centrifuged at 13,000× g for 10 min at 4 °C. The supernatant was then removed and placed into a clean 1.5 ml tube with half its volume of 100% ethanol. The tube was vortexed briefly before the contents underwent purification and DNase treatment, as detailed in the RNAqueous-Micro Kit manual. Purified DNAase-treated RNA was analyzed using a ND-1000 spectrophotometer (NanoDrop Technologies, Wilmington, DE) and a 2100 Bioanalyzer (Agilent Technologies, Santa Clara, CA), to determine the quantity and quality of the sample. RNA samples were used only if the A_260_/A_280_ ratio was above 1.9 and the RIN (RNA integrity number) was greater than 8.5.

### Affymetrix microarray analysis

The Affymetrix (Santa Clara, CA) Mouse Gene 1.0 ST Array was used; this is a whole transcript-based array that interrogates 28,853 well annotated genes. Staining, hybridization, washing, and scanning of the array were performed at the Biomolecular Resource Facility at the John Curtin School of Medical Research, Australian National University, following manufacturers’ protocols. Two experimental replicates were run independently, providing duplicates for each condition. Gene expression data were stored in .cel format files that could be interpreted by array analysis software.

### Quality assessment and hierarchical clustering

Affymetrix^®^ Expression Console™ software was used to supply probe set summarization and calculate quality assessment metrics. Gene expression data were uploaded into the Expression Console™ software to run a gene level core analysis. Once the analysis was complete, the numeric values of quality control metrics were summarized in a report and visualized in line graphs. Hierarchical clustering of data sets was performed using the heat map function in the Partek Genomics Suite (Partek Inc., St. Louis, MO). It uses log_2_ signal values. The following parameter settings were used: Method - average linkage; Dissimilarity - Euclidean; Clustering method-agglomerative.

### Statistical analysis

Gene expression files in .cel format were imported into the Partek Genomics Suite, with the following normalization of their configuration: Probes to import - interrogating probes; Probe filtering - include core; Background correction - RMA background; Log base - 2; Probe set summarization - Median Polish. Statistical differences between experimental groups were examined using a *t*-test (post-hoc contrasts).

### Gene expression in the inferior and superior regions of control retinas

Gene expression was compared between the superior and inferior regions of the control retinas using statistical and fold change analysis. In this comparison, there was no experimental procedure applied; therefore, the conventional term “fold change” seems inappropriate. Instead we use “fold difference.” Fold difference (FD) is then:

FD=E2/E1 when expression is greater in the second of two experimental (E) samples, FD=- E1/E2, when expression in the second sample is less.

In these equations, E1 is the level of expression in the first (usually the control) sample, and E2 is the level in the second (usually treated) sample. To apply this convention to the present samples, we chose the inferior retina as sample 2 (E2) and the superior retina as sample 1 (E1).

Data were then analyzed using two-sample *t*-tests for significant differences (increases and decreases) between the inferior and superior retina in the expression of all probes.

### Gene expression in the inferior and superior regions of hyperoxia-exposed retinas

To identify such genes, we analyzed the four samples arising from the two variables in this part of the study: treatment (hyperoxia versus control) and site (inferior versus superior). Each sample was available in duplicate. A 2-way ANOVA test applied to the eight data sets provided three p values for each gene, one giving the probability that the gene was not regulated by hyperoxia in the superior retina, the second giving the probability that the gene was not regulated in the inferior retina, and the third (the “combination” p value) giving the probability that the gene was not regulated in either region. For each gene, there were also available twofold ratio values, one that indicated the change in its expression induced by hyperoxia in the inferior retina, and a second that indicated the change in expression in the superior retina.

To identify genes regulated differently by hyperoxia in the two regions, we selected genes for which the 2-way ANOVA test gave a combination p value <0.05. Then we developed a specific measure of expression difference, called “fold margin” (FM).

We calculated a FM value for each gene, FM=FRi – FRs where FM is the fold margin, FRi is the fold ratio for that gene in the inferior retina, and FRs are the fold ratios for that gene in the superior retina.

### Functional analysis

Analysis of gene ontology and molecular pathways was performed using the bioinformatics resource Database for Annotation, Visualization and Integrated Discovery (DAVID) and Ingenuity Pathway Analysis (IPA) software (Ingenuity Systems Inc., Redwood City, CA). Using the Ingenuity Pathways Knowledge Base, IPA Core Analysis tools were used to assign genes to the most significant known molecular networks and biologic processes.

### Quantitative polymerase chain reaction

Quantitative PCR (qPCR) was used to validate the expression changes of selected genes identified in the microarray experiment. Three biologic replicates were used for each condition; RNA was processed as for the microarray experiments. For each sample, 1 µg retinal RNA was reverse transcribed into cDNA using the Superscript III Reverse Transcriptase protocol. The resulting cDNA sample served as a template for real time qPCR using TaqMan^®^ probes and accompanying Master Mix (Applied Biosystems, Foster City, CA). A typical 20 µl reaction is composed of 10 µl 2× TaqMan Gene Expression Master Mix, 1 µl TaqMan Gene Expression Assay, 0.4 µl cDNA templates and RNase-free water. All reactions were set up in duplicate and negative controls without any template or probe were included. The qPCR was performed on a Rotor-Gene 3000 and analyzed using the Rotor-Gene 6 software (Corbett Robotics, Mortlake, NSW, Australia). A typical qPCR program was used, including 2 min incubation at 50 °C and 10 min enzyme activation at 95 °C, followed by 40 cycles of 15 s denaturation at 95 °C plus 1 min probe annealing at 60 °C. The C_t_ (cycle threshold) means, representing the quantitation of the amount of cDNA in the original sample, were used to calculate fold change using the Pfaffl Equation [22]. Glyceraldehyde-3-phosphate dehydrogenase (*GAPDH)* was employed as a reference gene against which relative expression values of other genes were calculated.

## Results

### Clustering analysis

**Figure 1:**
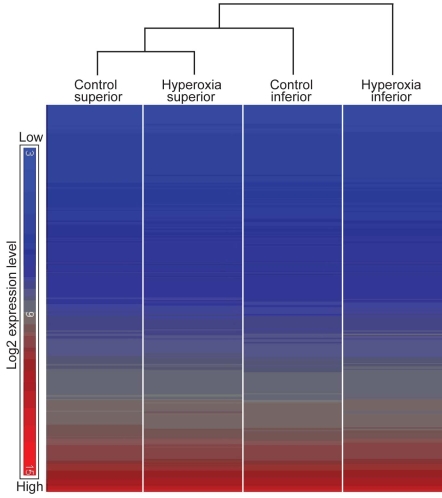
Hierarchical clustering diagram. The study aims to identify the genes expressed differently in normoxic (control) and hyperoxic conditions in the superior and inferior halves of the retina because regional sensitivity to hyperoxia of the inferior was expected. Clustering analysis demonstrated similarity and correlation between replicates within an experimental group and between different groups. Each column comprises a set of horizontal lines with each line representing a single gene or noncoding RNA (ncRNA). The color given to the line depends on the level of expression of the gene, from blue (low expression) to red (high expression); the range of colors available is shown at left. In each column, the genes are arranged in low-to-high expression, going down the column. The program then compares the order of genes in the column and indicates, by the brackets at top, the similarities and differences between the four samples. The replicates of each sample clustered closely to each other (not shown) and were averaged to constitute the four samples shown. The samples from the superior retina before and after hyperoxia were most similar. The sample from the inferior retina after hyperoxia, in which photoreceptor degeneration was greatest, is most dissimilar from the other samples.

Hierarchical clustering analysis was performed using the heat map function in the Partek Genomics Suite (Figure 1). The replicates of each group clustered most closely to each other (GEO accession number GSE21246) and were therefore averaged; Figure 1 therefore shows the clustering of the averaged samples. Superior retina samples before and after hyperoxia clustered more closely to each other than to other groups, indicating that hyperoxia had little effect on the superior retina. Gene expression in the inferior retina diverged from the superior retina, with hyperoxia increasing the separation.

### Control retina: genes differently expressed in inferior and superior regions

Comparison of gene expression levels in inferior and superior regions of control retinas identified expression changes in a limited cohort of sequences.

### Identification

The FD analysis described in the Methods section identified only 31 sequences with an FD >1.5. Of these, 27 (listed in Table 1) are known genes. Approximately half of the genes were expressed more highly in the inferior retina, and the other half were expressed more highly in the superior retina. The FD was greater than two for only two of the 27 genes (T box 5 [*Tbx5],* membrane metalloendopeptidase *[Mme]).*

**Table 1:** Differentially expressed genes between inferior and superior retina of control (normoxic) C57BL/6J mice.

**Symbol**	**Description**	**RefSeq**	**p value**	**FD (I/S)**
**Anatomic structure development (GO:0009653)**				
Vax2	ventral anterior homeobox containing gene 2	NM_011912	0.021	1.840
Sprr2b	small proline-rich protein 2B	NM_011469	0.012	1.630
Vcan	versican	NM_001081249	0.001	1.598
Slc26a4	solute carrier family 26, member 4	NM_011867	0.008	1.598
Nr2f2	nuclear receptor subfamily 2, group F, member 2	NM_009697	0.002	−1.564
Efnb2	ephrin B2	NM_010111	0.015	−1.625
Tbx5	T-box 5	NM_011537	0.0002	−2.087
**Sensory perception (GO:0007600)**				
Opn1sw	opsin 1 (cone pigments), short-wave-sensitive	NM_007538	0.038	1.762
V1rb8	vomeronasal 1 receptor, B8	NM_053229	0.043	−1.508
Olfr1416	olfactory receptor 1416	NM_147038	0.041	−1.577
Opn1mw	opsin 1 (cone pigments), medium-wave-sensitive	NM_008106	0.010	−1.644
Olfr652	olfactory receptor 652	NM_147048	0.017	−1.652
**Catalytic activity**				
Mme	membrane metalloendopeptidase	NM_008604	0.031	2.197
Adh6b	alcohol dehydrogenase 6B (class V)	ENSMUST00000090166	0.017	1.516
Rdh16	retinol dehydrogenase 16	NM_009040	0.004	−1.684
Alpk2	alpha-kinase 2	ENSMUST00000035548	0.017	−1.979
**G protein-coupled receptor**				
Gpr137b	G protein-coupled receptor 137B	NM_031999	0.007	1.541
Gprc5b	G protein-coupled receptor C5B	NM_022420	0.025	1.526
**Amino acid transporter**				
Slc7a11	solute carrier family 7	NM_011990	0.035	1.756
**Cell migration**				
Sst	somatostatin	NM_009215	0.016	1.666
**Translation regulation**				
Igf2bp2	insulin-like growth factor 2 mRNA binding protein 2	NM_183029	0.024	−1.544
**Inductin of apoptosis**				
Khdc1a	KH domain containing 1A	NM_183322	0.037	−1.575
**Unknown function**				
Snhg1	small nucleolar RNA host gene 1	AK051045	0.007	1.710
	predicted gene, EG624256	XR_034994	0.006	1.529
	predicted gene, EG668725	DQ386867	0.039	1.510
Abi3bp	ABI gene family, member 3 (NESH) binding protein	NM_001014423	0.028	1.505
	RIKEN cDNA A530032D15Rik gene	BC094285	0.007	−1.503
Gm1381	gene model 1381, (NCBI)	BC147167	0.005	−1.523
Serpina1b	serine preptidase inhibitor, clade A member 1B	NM_009244	0.025	−1.557
Gm1862	gene model 1862, (NCBI)	XM_001478226	0.031	−1.576
	predicted gene, OTTMUSG00000001136	XM_001005073	0.007	−1.868

Genes were grouped into several functional categories using gene ontology analysis. Categories with GO number are considered specific functional groups. Abbreviations: RefSeq represents NCBI reference sequence; FD represents fold difference; GO represents gene ontology.

### Functional analysis

The list of 27 genes was subjected to gene ontology analysis, using the bioinformatics resource DAVID, which organized the genes into several functional groups, including Anatomic structure development, Sensory perception, and Catalytic activity (Table 1). Genes related to retinal development formed the largest functional group; these relate to the organization of the retina’s projection to brain centers. A superior-inferior gradient is consistent with previous work on these genes [23-25]. Five of the 27 genes were in the Sensory perception group. Two genes are for short- and medium-wavelength cones; short-wave-sensitive cone opsin1 (*Opsn1sw*) is more strongly expressed in the inferior retina, and medium-wave-sensitive cone opsin1 (*Opsn1mw*) in the superior retina. These differences in expression match known regional differences in the retinal distributions of short- and medium-length cones [26-28], and confirm the present separation of inferior and superior retina samples.

### Quantitative PCR validation

**Figure 2:**
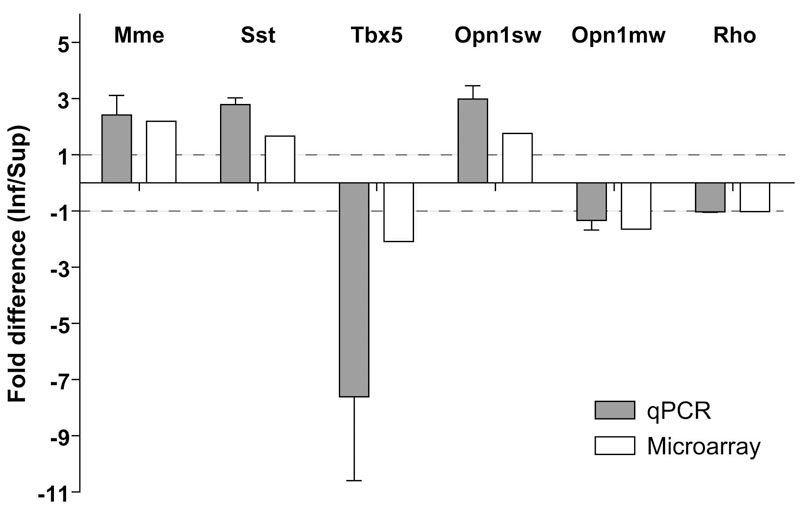
Quantitative PCR for microarray validation. Array validation was performed by quantitative RT–PCR for 5 genes selected from the list in Table 1. Rhodopsin was used as a retina-relevant reference gene. qPCR is found in excellent agreement with the outcome of microarray analysis. The results of qPCR were averaged from three independent experiments, with the error bar indicating the standard error of the mean. Dashed lines at fold differences of 1 and −1 indicate no difference of gene expression between the inferior and superior regions.

Six genes were selected for confirmation of their differential expression in control retinas, using qPCR (Figure 2). The gene *Tbx5* was chosen as an example of the Anatomic structure group, *Opn1sw* and *Opn1mw* were chosen as retina-relevant examples from the Sensory perception group, *Mme* and somatostatin (*Sst)* were chosen as a functionally inter-related pair, and rhodopsin *(Rho)* was chosen as a non-regulated, retina-relevant gene. Quantitative PCR confirmed the even expression of *Rho* and the differential regulation of *Mme, Sst, Tbx5,* and *Opn1sw*. For one gene, *Opn1mw* (encoding M-opsin), the FD assessed by qPCR was less than the threshold of 1.5 fold (−1.33 by PCR versus −1.64 by microarray). However, the difference was in the same direction in the qPCR and microarray assessments.

### Hyperoxia-exposed retina: genes differently regulated in inferior and superior regions

Genes whose regulation by hyperoxia differed significantly between the inferior and superior retina could include genes regulated in one region but not the other, genes upregulated in one region and downregulated in the other, or genes regulated in the same direction in the two regions, but much more strongly in one region.

### Identification

**Figure 3:**
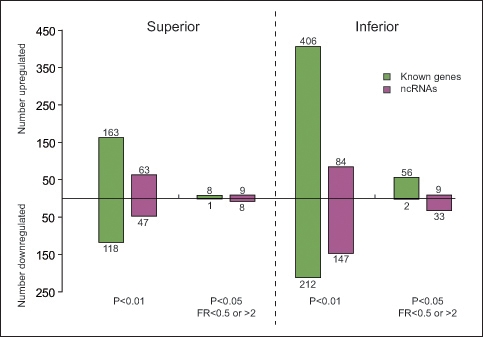
Significant more genes regulated in the inferior than the superior retina after hyperoxia. According to the different selection criteria, the number of genes and non-coding RNAs regulated by hyperoxia were assessed and separately elucidated for the superior and inferior regions. Fold ratio (FR) values < 0.5 indicate significant downregulation of expression (at least halved); FR values > 2 indicate significant upregulation of expression (more than doubled).

Hyperoxia induced significant changes in gene expression in both the inferior (hyperoxia-vulnerable) and superior (hyperoxia-resistant) retina, but the number of hyperoxia-regulated genes was much higher in the inferior retina (Figure 3). For example, using the criterion p<0.01 on the 2-way ANOVA test, the expression of 281 genes and 110 ncRNAs changed significantly in the superior retina, while the expression of 618 genes and 231 ncRNAs changed in the inferior retina. Using p≤0.05 and requiring a fold ratio > 2 or < 0.5, the total number of genes differentially expressed was over six times greater in the inferior retina than in the superior retina (58 versus 9). Overall, more genes were upregulated than downregulated. This imbalance was particularly marked after applying both selection criteria; for example, 56 genes were upregulated in the inferior retina, compared to 2 that were downregulated.

Combining the criteria of p<0.05 and abs (FM) >0.5 (Table 2), a total of 112 sequences with Affymetrix probe IDs were identified as differently regulated by hyperoxia between the inferior and superior regions. Of these 112, 97 matched known genes (Table 2). The gene with the most significant FM was *Edn2* (endothelin 2), a possible stress response gene. The expression of gene *Edn2* increased 7.02 fold in the inferior retina, but increased just 1.62 fold in the superior retina after being exposed to hyperoxia.

**Table 2:** Known genes and ncRNAs differentially regulated by hyperoxia between inferior and superior retina.

**Symbol**	**Description**	**RefSeq**	**P-value** Treat*Site	**FR** **Inf**	**FR** **Sup**	**FM**
Edn2	endothelin 2	NM_007902	0.00001	7.02	1.62	5.40
Gfap	glial fibrillary acidic protein, mRNA	NM_010277	0.00303	5.36	2.37	2.99
C1qb	complement component 1, beta polypeptide	NM_009777	0.00085	4.20	1.45	2.75
Bcl3	B-cell leukemia/lymphoma 3	NM_033601	0.00846	3.89	1.48	2.42
C1qc	complement component 1, subcomponent, C chain	NM_007574	0.00468	4.21	1.93	2.28
Mpeg1	macrophage expressed gene 1	NM_010821	0.00848	3.83	1.57	2.27
C1qa	complement component 1, subcomponent, alpha polypeptide	NM_007572	0.01737	3.69	1.68	2.01
Cd68	CD68 antigen	NM_009853	0.01188	3.27	1.45	1.83
Ly86	lymphocyte antigen 86	NM_010745	0.00329	3.95	2.16	1.78
Fgf2	fibroblast growth factor 2	NM_008006	0.00038	3.10	1.45	1.64
Timp1	tissue inhibitor of metalloproteinase 1	NM_001044384	0.00172	2.74	1.14	1.61
Fcrls	Fc receptor-like S, scavenger receptor	NM_030707	0.00557	2.55	1.02	1.53
Laptm5	lysosomal-associated protein transmembrane 5	NM_010686	0.00863	3.03	1.58	1.45
Ccl3	chemokine (C-C motif) ligand 3	NM_011337	0.01929	2.46	1.06	1.40
Ctss	cathepsin S	NM_021281	0.00330	3.03	1.68	1.35
Lrrc2	leucine rich repeat containing 2	NM_028838	0.00324	2.71	1.48	1.23
Crym	crystallin, mu	NM_016669	0.02834	2.74	1.52	1.22
Slc25a37	solute carrier family 25, member 37	NM_026331	0.00087	2.43	1.21	1.22
Prtg	protogenin homolog (Gallus gallus)	NM_175485	0.00036	2.35	1.19	1.16
Lad1	ladinin	NM_133664	0.00227	2.36	1.20	1.16
Cst7	cystatin F (leukocystatin)	NM_009977	0.03019	2.07	0.95	1.12
Fcer1g	Fc receptor, IgE, high affinity I, gamma polypeptide	NM_010185	0.00232	2.44	1.33	1.11
Gadd45b	growth arrest and DNA-damage-inducible 45 beta	NM_008655	0.01173	2.74	1.65	1.09
Chi3l1	chitinase 3-like 1	NM_007695	0.02641	2.88	1.84	1.04
Cd180	CD180 antigen	NM_008533	0.02177	2.10	1.07	1.02
Mt2	metallothionein 2	NM_008630	0.03497	2.41	1.39	1.02
Klhl29	kelch-like 29 (Drosophila)	BC145748	0.00162	2.20	1.18	1.02
Socs3	suppressor of cytokine signaling 3	NM_007707	0.00892	2.12	1.13	1.00
Cd53	CD53 antigen	NM_007651	0.01901	2.35	1.36	0.99
Olfr43	olfactory receptor 43	NM_146711	0.03715	1.77	0.79	0.99
S100a6	S100 calcium binding protein A6 (calcyclin)	NM_011313	0.03199	2.06	1.08	0.98
Tyrobp	TYRO protein tyrosine kinase binding protein	NM_011662	0.00115	2.25	1.27	0.98
Ctla2a	cytotoxic T lymphocyte-associated protein 2 alpha	NM_007796	0.04101	1.88	0.91	0.97
Csf1r	colony stimulating factor 1 receptor	NM_001037859	0.00009	2.37	1.40	0.97
Stat3	signal transducer and activator of transcription 3	NM_213659	0.00102	2.40	1.44	0.96
Antxr2	anthrax toxin receptor 2	NM_133738	0.00279	2.23	1.28	0.95
C3ar1	complement component 3a receptor 1	NM_009779	0.01664	1.96	1.02	0.95
Kremen1	kringle containing transmembrane protein 1	NM_032396	0.00444	2.02	1.07	0.95
C3	complement component 3	NM_009778	0.03059	2.23	1.28	0.94
B2m	beta-2 microglobulin	NM_009735	0.00093	2.38	1.44	0.94
Lcn2	lipocalin 2	NM_008491	0.00238	2.13	1.19	0.94
Irgm1	immunity-related GTPase family M member 1	NM_008326	0.02159	2.04	1.12	0.93
Khdc1a	KH domain containing 1A	NM_183322	0.02946	1.73	0.87	0.86
Chl1	cell adhesion molecule with homology to L1CAM	NM_007697	0.01079	1.78	0.92	0.86
H2-D1 (HLA-C)	histocompatibility 2, D region locus 1	NM_010380	0.00124	2.00	1.16	0.84
H2-K1	histocompatibility 2, K1, K region	NM_001001892	0.00681	2.05	1.22	0.83
Osmr	oncostatin M receptor	NM_011019	0.01265	2.70	1.91	0.79
Fcgr3 (Fcrg2a)	Fc receptor, IgG, low affinity III	NM_010188	0.01391	1.79	1.01	0.78
Rbm17	RNA binding motif protein 17	NM_152824	0.00686	1.88	1.10	0.78
H2-Q7	histocompatibility 2, Q region locus 7	NM_010394	0.00093	1.89	1.14	0.75
Lin7b	lin-7 homolog B (C. elegans)	NM_011698	0.00908	1.83	1.11	0.72
Irf9	interferon regulatory factor 9	NM_008394	0.00479	2.21	1.50	0.70
Gnb3	guanine nucleotide binding protein, beta 3	NM_013530	0.00035	1.83	1.14	0.69
Spp1	secreted phosphoprotein 1	NM_009263	0.00216	1.77	1.10	0.68
Moxd2	monooxygenase, DBH-like 2	NM_139296	0.01505	1.58	0.94	0.65
Agtpbp1	ATP/GTP binding protein 1	NM_023328	0.00242	1.76	1.12	0.64
Olfr652	olfactory receptor 652	NM_147048	0.02679	1.38	0.74	0.64
Ly9	lymphocyte antigen 9	NM_008534	0.02946	1.59	0.95	0.64
Myo10	myosin X	NM_019472	0.00714	1.94	1.31	0.63
V1rb8	vomeronasal 1 receptor, B8	NM_053229	0.04461	1.42	0.80	0.62
Sec22c	SEC22 vesicle trafficking protein-like C (S. cerevisiae)	NM_178677	0.01512	1.65	1.04	0.61
Sulf2	sulfatase 2	NM_028072	0.03293	1.58	0.97	0.61
Cd33	CD33 antigen	NM_001111058	0.00293	1.63	1.03	0.60
Hexb	hexosaminidase B	NM_010422	0.00341	1.88	1.30	0.58
Pld4	phospholipase D family, member 4	NM_178911	0.01563	1.84	1.27	0.57
Skap2	src family associated phosphoprotein 2	NM_018773	0.00550	1.84	1.27	0.57
Usp18	ubiquitin specific peptidase 18	NM_011909	0.02940	1.58	1.02	0.56
Vmn2r68	vomeronasal 2, receptor 68	NM_001105181	0.00628	1.47	0.92	0.56
Bst2	bone marrow stromal cell antigen 2	NM_198095	0.00535	1.60	1.05	0.55
Wfs1	Wolfram syndrome 1 homolog (human)	NM_011716	0.02286	1.54	0.99	0.54
Rdh16	retinol dehydrogenase 16	NM_009040	0.00992	1.25	0.72	0.53
V1rd6	vomeronasal 1 receptor, D6	NM_030738	0.04247	1.30	0.77	0.53
Shbg	sex hormone binding globulin	NM_011367	0.01306	1.47	0.94	0.53
H2-Q2 (HLA-F)	histocompatibility 2, Q region locus 2	AB359227	0.00277	1.71	1.18	0.53
Sulf1	sulfatase 1	NM_172294	0.00558	1.68	1.16	0.52
Atf3	activating transcription factor 3	NM_007498	0.02131	1.63	1.11	0.52
Mt1	metallothionein 1	NM_013602	0.00379	1.57	1.06	0.51
V1re6	vomeronasal 1 receptor, E6	NM_134195	0.01502	1.37	0.86	0.51
Dcps	decapping enzyme, scavenger	NM_027030	0.02157	1.56	1.06	0.50
Tead3	TEA domain family member 3	NM_011566	0.01222	1.66	1.16	0.50
Bmp2	bone morphogenetic protein 2	NM_007553	0.03978	0.71	1.21	−0.50
Defb9	defensin beta 9	NM_139219	0.01100	0.81	1.32	−0.51
Slc6a13	solute carrier family 6 (neurotransmitter transporter, G protein), member a13	NM_144512	0.02354	0.92	1.43	−0.51
Ankrd44	ankyrin repeat domain 44	NM_001081433	0.02845	0.91	1.42	−0.51
Clec14a	C-type lectin domain family 14, member a	NM_025809	0.01571	0.88	1.40	−0.52
Colec12	collectin sub-family member 12	NM_130449	0.00944	0.85	1.37	−0.52
Wnt8a	wingless-related MMTV integration site 8A	NM_009290	0.00618	0.86	1.39	−0.53
Kcnq5	potassium voltage-gated channel, subfamily Q, member 5	NM_023872	0.01549	0.82	1.36	−0.54
Suclg2	succinate-CoA ligase, GDP-forming, beta subunit	NM_011507	0.04602	0.86	1.42	−0.55
Slc16a12	solute carrier family 16 member a12	NM_172838	0.01174	0.77	1.33	−0.56
Snhg1	small nucleolar RNA host gene 1	AK051045	0.00764	0.52	1.08	−0.56
Vcan	versican	NM_001081249	0.00164	0.91	1.48	−0.57
Abpb	androgen binding protein beta	NM_001100464	0.02420	0.79	1.36	−0.57
Mitf	microphthalmia-associated transcription factor	NM_001113198	0.00183	0.79	1.38	−0.58
Nr2e3	nuclear receptor subfamily 2, group E, member 3	NM_013708	0.00803	0.84	1.45	−0.61
Josd3	Josephin domain containing 3	BC056964	0.01926	0.64	1.28	−0.64
Slc26a4	solute carrier family 26, member 4	NM_011867	0.00514	0.71	1.48	−0.77
OTTMUSG00000007392		ENSMUST00000068009	0.02400	2.63	1.11	1.52
ENSMUST00000105041		ENSMUST00000105041	0.00238	2.31	1.20	1.11
LOC546230		ENSMUST00000103543	0.04064	2.04	1.06	0.99
OTTMUSG00000001136		XM_001005073	0.01136	1.33	0.63	0.71
A530032D15Rik		BC094285	0.00291	1.32	0.64	0.69
BC094916		NM_001024721	0.04309	1.09	0.46	0.64
A630098G03Rik		EU099309	0.03236	1.34	0.74	0.60
LOC544737		AK143218	0.02940	1.33	0.79	0.54
1110054P19Rik		NM_026834	0.01399	1.36	0.85	0.51
EG668103		XM_994312	0.01637	1.37	0.87	0.50
ENSMUSG00000071362		ENSMUST00000072014	0.01789	0.73	1.23	−0.50
ENSMUST00000054825		ENSMUST00000054825	0.01130	0.66	1.23	−0.56
ENSMUSG00000073593		ENSMUST00000097611	0.03139	0.73	1.30	−0.57
EG435337		NM_001013824	0.04833	0.66	1.26	−0.60
E430024C06Rik		AK149411	0.04984	0.45	1.32	−0.87

The selection of genes was made using the criteria p<0.05 and abs(FM) > 0.5. They are sorted by the value of fold margin. Unknown sequences were listed separately, following the known genes. Abbreviations: FR represents fold ratio; FM represents fold margin; inf represents inferior; sup represents superior; RefSeq represents NCBIreference sequence.

### Functional analysis

Ingenuity Pathway Analysis software was used to identify biologic functions, diseases, and well characterized pathways that are most relevant to the genes of interest. The program compared the genes identified as differentially regulated between the inferior and superior retina with a database of genes involved in particular functions, diseases, and pathways. It then calculated the probability that each function, disease, or pathway is not involved in the differential regulation. Using the criteria p<0.001 and a minimum involvement of five genes, the processes and diseases listed in Table 3 were identified. Some clusters of differentially regulated genes that possibly impact photoreceptor vulnerability and survival are listed in Table 4, arranged according to function, disease, or pathway.

**Table 3:** The “Biologic processes” and “Disease and disorder” groupings of genes identified as differentially expressed between superior and inferior retina after exposure to hyperoxia.

**Relevant Functions and Diseases**	**Number of molecules**	**p-value**
**Biologic process**		
defense response	22	5.9E-11
immune response	19	2.7E-10
response to external stimulus	13	7.9E-06
immune effector process	8	1.1E-05
regulation of multicellular organismal process	10	2.8E-05
antigen processing and presentation	7	4.0E-05
regulation of immune system process	7	4.6E-05
activation of immune response	6	7.1E-05
response to stress	14	3.2E-04
**Disease and disorder**		
immunological disorder	19	1.5E-08
inflammatory disorder	21	3.1E-08
neurologic disorder	26	2.6E-07
genetic disorder	38	1.1E-06
infectious disorder	10	5.7E-05
respiratory disorder	8	1.2E-04
ophthalmic disorder	7	5.1E-04

The two groupings shown met the criteria of significant relevance (p<0.001) and a minimum involvement of 5 genes from gene list of interest (Table 2).

**Table 4:** Clusters of genes differentially regulated between inferior and superior retina, with possible impact on photoreceptor vulnerability and survival.

**Symbol**	**Description**	**RefSeq**	**FR (inf)**	**FR (sup)**	**FM**
**Complement system (p=1.66E-07)**					
C1qb	complement component 1, q subcomponent, beta polypeptide	NM_009777	4.20	1.45	2.75
C1qc	complement component 1, q subcomponent, C chain	NM_007574	4.21	1.93	2.28
C1qa	complement component 1, q subcomponent, alpha polypeptide	NM_007572	3.69	1.68	2.01
C3ar1	complement component 3a receptor 1	NM_009779	1.96	1.02	0.95
C3	complement component 3	NM_009778	2.23	1.28	0.94
**Acute phase response signaling, IL-10 signaling (p=1.8E-04–4.07E-03)**					
Bcl3	B-cell leukemia/lymphoma 3	NM_033601	3.89	1.48	2.42
Socs3	suppressor of cytokine signaling 3	NM_007707	2.12	1.13	1.00
Stat3	signal transducer and activator of transcription 3	NM_213659	2.40	1.44	0.96
**Ophthalmic disorders (p=5.10E-04)**					
Timp1	tissue inhibitor of metalloproteinase 1	NM_001044384	2.74	1.14	1.61
Ccl3	chemokine (C-C motif) ligand 3	NM_011337	2.46	1.06	1.40
C3ar1	complement component 3a receptor 1	NM_009779	1.96	1.02	0.95
C3	complement component 3	NM_009778	2.23	1.28	0.94
Slc16a12	solute carrier family 16 (monocarboxylic acid transport)	NM_172838	0.77	1.33	−0.56
Vcan	versican	NM_001081249	0.91	1.48	−0.57
Nr2e3	nuclear receptor subfamily 2, group E, member 3	NM_013708	0.84	1.45	−0.61
**Oxidative stress response (p=1.66E-04)**					
Mt2, Mt1e	metallothionein 2	NM_008630	2.41	1.39	1.02
Mt1, Mt1f	metallothionein 1	NM_013602	1.57	1.06	0.51

P value accounts for the probability that each function, disease or pathway is not involved in the differential regulation. Abbreviations: FR represents fold ratio; FM represents fold margin; inf represents inferior; sup represents superior; RefSeq represents NCBI reference sequence.

Interestingly, four of the biologic processes identified in Table 3 are strongly related to the function of the immune system. Moreover, “immunological disorders” is the disease category most strongly related to the gene set identified as differentially regulated by hyperoxia between the inferior and superior retina.

### Quantitative PCR validation

Expression changes of 11 genes were selected for confirmation by qPCR. From these, eight genes were chosen from Table 2: *Edn2*, glial fibrillary acidic protein (*Gfap)*, B-cell leukemia/lymphoma 3 (*Bcl-3)*, nuclear receptor subfamily 2, group E, member 3 (*Nr2e3)*, wingless-related MMTV integration site 8A (*Wnt8a)*, lin-7 homolog B (*Lin7a)*, complement component 3 (*C3),* and growth arrest and DNA-damage-inducible 45 beta (*Gadd45b)*. Three other genes were also chosen for validation: cyclic nucleotide gated channel alpha 2 (*Cnga2*), which has a p-value <0.05 but an FM <0.5, hypoxia inducible factor 1 alpha subunit (*Hif1α*), which has shown responses to hypoxia in previous studies [29,30], and medium-wave-sensitive cone opsin (*Opn1mw*), which is a gene known to be expressed by the retina and that is not hyperoxia-regulated [14].

**Figure 4:**
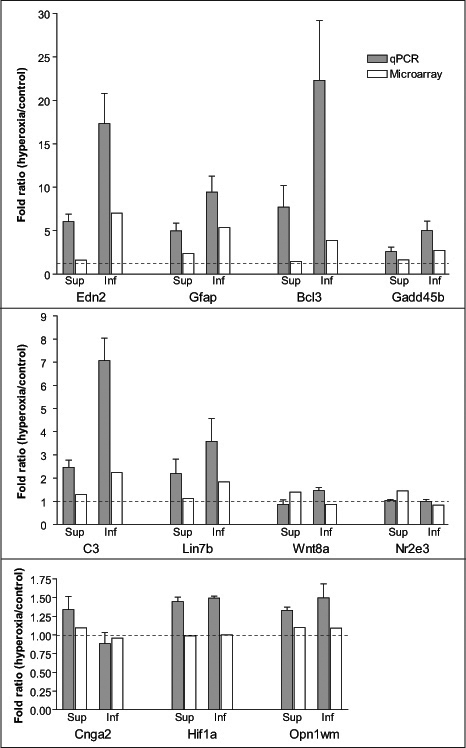
Quantitative PCR validation of expression changes induced by exposure to hyperoxia in a selected cohort of genes. For each gene, the change was estimated in both the superior and inferior retina by microarray analysis and qPCR. The results of qPCR were averaged from three independent experiments, with the error bar indicating the standard error of the mean. Fold ratio values >1 indicate an upregulation and values <1 indicate a downregulation. Where FR=1 (dotted line), expression was unchanged.

The regulation changes of these genes observed by qPCR and in the microarray are shown in Figure 4. The direction of the change was confirmed in six of the eight genes that differentially regulate between the inferior and superior retina; the exceptions were *Wnt8a* and *Nr2e3*. The magnitude of the FM between the superior and inferior retina was more dramatic for genes *Edn2*, *Gfap,* and *Bcl3*. For *Cnga2*, the upregulation in the superior and downregulation in the inferior retina were more noticeable; however, the FM between them was < 0.5, which was consistent with the microarray data. For *Hif1α* and *Opn1mw,* which showed no significant change in the microarray, upregulation of approximately 30%–50% was observed by qPCR. However, such upregulation showed no regional differences. Upregulation of *Hif1α* by hyperoxia has been reported previously [14], but the upregulation was time-dependent, being prominent after three days of exposure, but not at the 14-day exposure used here.

## Discussion

The microarray analysis presented provides an overview of regional variations in gene expression in the normal retina before and after hyperoxic stress. Further, immunohistochemical studies will be needed to identify the sites at which gene products are expressed. Although hyperoxia is specifically toxic to photoreceptors, hyperoxia may regulate genes in any of the several classes of retinal neurons and neuroglia, and in the endothelium of blood vessels.

### Gene expression before hyperoxia

Comparing gene expression between the superior and inferior retina under normoxic (control) conditions, two genes showed an FD in expression of greater than two, and 27 showed an FD of greater than 1.5. These differences in gene expression are relatively limited, but may be significant in determining the relative invulnerability of the superior retina to hyperoxic damage and, conversely, the vulnerability of the inferior retina.

Seven out of the 27 genes (listed in Table 1) are involved in anatomic structure development (gene ontology GO-term 0009653). Vax2, Tbx5, and ephrin B2 (Efnb2) are important in eye morphogenesis and are topographic determinants of the visual projections between the retina and tectum. In vertebrate development, the polarity of the dorsal-ventral (dorsal-superior, ventral-inferior) eye axis is determined by spatially restricted expression of transcription factors Vax2 in the inferior retina and Tbx5 in the superior retina [24,25]. Efnb2 is an axon guidance molecule expressed in a low-to-high superior-inferior gradient in the ganglion cell layer [31]. The expression of Efnb2 and its receptor is regulated by Vax2 and Tbx5, which in turn control the dorsal-ventral polarity of retinotectal projection [23]. These developmental patterns were detected in the adult retina in the present experiments, providing evidence that our separation of inferior-superior retina was accurate.

Five genes are associated with sensory perception (GO-term 0007600). They belong to the rhodopsin-like superfamily of G-protein-coupled receptors, although they are involved in distinct sensory systems. *Opn1mw* encodes the medium-wave-sensitive cone opsins (expressed in M-cones) and *Opn1sw* encodes the short-wave-sensitive cone opsins (S-cones or “blue” cones). The uneven distribution of cone receptors, with S-cones more numerous in the inferior retina and M-cones in superior retina, has been described in mice [27,28] and cats [26]. The expression differences observed (1.76 for *Opn1sw* in an inferior-superior comparison and −1.64 for *Opn1mw*) confirm the differences in cone topography detected by immunohistochemistry.

The gene showing the largest FD between the superior and inferior regions of the normoxic retina is *Mme*, which is expressed more than twofold in the inferior retina. The product of this gene is a membrane-bound metalloendopeptidase, commonly known as neutral endopeptidase or neprilysin. It has been found in various cell lines, including endothelial cells and vascular smooth muscle cells. Mme belongs to a family of zinc metalloproteases [32].

Another member of this family is endothelin converting enzyme-1 (ECE-1), which shares 58% amino acid homology with Mme [33]. ECE-1 catalyzes big endothelin to peptide endothelin and plays a key role in proteolytic endothelin activation [34]. However, the conversion of big endothelins to their respective endothelin peptides may not solely depend on ECE-1. In guinea pig lung parenchyma, Mme was suggested to play a role in the conversion to endothelin-1 and endothelin-2 [35]. Given their structural homology, it is not surprising that they share functions. As reported previously [14], endothelin-2 is the most upregulated gene in the whole retina after two weeks of hyperoxia. In the present study, endothelin-2 is the most upregulated gene in the vulnerable inferior retina after hyperoxic exposure. This correlates with the overexpression of Mme in the inferior retina. Perhaps Mme is responsible for or at least represents one pathway of the biosynthesis of endothelin-2 in the inferior retina after hyperoxic exposure.

Somatostatin is uniquely capable of stimulating Mme activity in primary cortical neurons [36]. Most interestingly, the gene encoding somatostatin (*Sst*) is also expressed more in the inferior than the superior retina (Table 1). The relatively high expression of both *Mme* and *Sst* may provide some insight into the vulnerability of the inferior mouse retina. One possibility is that there is an unknown regulatory peptide that protects photoreceptors against oxidative damage and is also, like amyloid beta (Aβ), a substrate of Mme. Upregulation of Mme might degrade this peptide, reduce its protective action, and cause the vulnerability of the inferior retina to hyperoxic stress. Other possibilities include regional variations in retinal metabolism, which in turn may be observed in regional variations in retinal blood supply. In the rabbit and monkey, for example, regional variations in the choroidal circulation (the major source of oxygen supply to photoreceptors) have been described, matching retinal specializations [37]. An inferior-superior difference in the retinal circulation has also been observed in humans [38].

Another established difference between the superior and inferior retina is environmentally determined. When animals are raised (as in the present experiments) in light from sources in the ceiling of a room, the inferior retina is more exposed to ambient light and is more resistant to subsequent light stress [17]. Exposure to modest ambient light can “pre-condition” the retina, making it resistant to subsequent hyperoxic stress [39]. However, the relative vulnerability of the inferior retina clearly cannot be explained by such a mechanism. The current results provide a starting point for further detailed study and are a first step in describing the mechanisms involved.

### Gene expression after hyperoxia

#### Comparison with previous microarray studies

The present data show that hyperoxia-induced regulation of genes occurred significantly more in the inferior retina in the C57BL/6J mouse retina (Figure 3); the number of significantly regulated known genes identified was approximately sevenfold greater in the inferior retina (58 versus nine). The present data can be compared to the all-retina study of the Natoli group [14], who reported that following hyperoxic exposure for 14 days, 77 known genes were significantly regulated, using the same fold change criterion. Of the 9 genes significantly regulated by hyperoxia in the superior retina in the present study, five (55%) are also on Natoli et al. and coworkers list of all-retina regulated genes. Of the 58 genes significantly regulated by hyperoxia in the inferior retina, 26 (45%) are also on the list of all-retina regulated genes. It was also of interest to compare the inferior-specific regulated genes (Table 2, 97 known genes) with those identified by Natoli group as hyperoxia-regulated in their all-retina analysis. Of the 77 genes identified by Natoli group, 21 (27%) were also in the inferior-specific list, including *Bcl3, C3, Edn2, Gfap,* and *Gadd45b*.

One previous microarray study has described the genes regulated by light damage in mice using the light-vulnerable BALB/cJ mouse strain [40]. The authors of that study reported that 70 genes were upregulated at least twofold. Fourteen of these 70 genes (20%) also displayed differential expression change in this study. It is possible that the light vulnerability of retina in BALB/cJ mice and the hyperoxia vulnerability of the inferior retina of C57BL/6J mice share some common features and mechanisms.

### Endothelin-2

The gene *Edn2* that encodes endothelin-2 is the most strongly regulated gene in the inferior retina after hyperoxic exposure and is also the most differentially regulated gene between the inferior and superior retina after hyperoxic exposure (Table 2). Its expression increased sevenfold in the inferior retina compared to 1.6 fold in the superior retina after exposure to hyperoxia.

Endothelin-2 belongs to a family of peptides comprising endothelin-1, endothelin-2, and endothelin-3 [41]. The endothelins are stress-responsive regulators that work in paracrine and autocrine fashion in a variety of organs, with either beneficial or detrimental results. For example, endothelin-1 is a potent vasoconstrictor in blood vessels. It also acts as a mitogen on the vascular smooth muscle and has been implicated in several vascular diseases, such as hypertension and atherosclerosis [42].

Endothelin-2 has been found to be produced in endothelial cells, the heart, and the kidneys [43,44]. In the retina, the expression of Edn2 is highly induced in models of retinitis pigmentosa, retinal detachment, and light damage and the increased production of Edn2 is localized to photoreceptors [45]. Photoreceptor-derived Edn2 is suggested to serve as a stress signal in the signaling cascade between the photoreceptor and Müller glia cells. In the presence of damaging stress such as excessive light exposure and gene mutation, Müller glia cells sense the photoreceptor damage and react by upregulating leukemia inhibitory factor (LIF) [46]. An increase in leukemia inhibitory factor in turn induces the expression of Edn2 in photoreceptors. The signal is then passed back to Müller glia cells by binding Edn2 to endothelin receptor B (Ednrb), which is expressed on Müller cells. This positive feedback loop then stimulates the production of survival factors, such as fibroblast growth factor 2 (FGF2), in Müller cells and/or photoreceptors to protect visual cells from further damage [45,46].

### Involvement of the immune system

Misregulation of the innate immune response and other immune-mediated processes has been suggested to play a role in the development and progression of retinal degenerative diseases, such as age-related macular degeneration (AMD), in human patients [47,48]. The involvement of inflammation and immune-mediated processes has been suggested, for example, in the formation of drusen and inflammation triggered by the debris generated by dying retinal pigment epithelial (RPE) cells, as a factor in photoreceptor cell death [48].

Complement cascade proteins have been shown to be upregulated in the AMD retina, showing a strong presence along the RPE-choroid interface and in drusen, the hallmark of AMD, suggesting that the activation of the complement pathway has a potential role in drusen biogenesis and the etiology of AMD [47]. A murine model of laser-induced choroidal neovascularization (CNV) in C57BL/6J mice revealed the deposition of C3 and membrane attack complex in the neovascularizing tissue. Complement depletion in C3^−/−^ mice resulted in the inhibition of CNV and a marked reduction of angiogenic factors [49]. C3a is the bioactive fragment of complement component C3; a blockade of the C3a receptors (C3aR) also reduces CNV [50]. In addition, in the degenerative mouse model *rd1*, the gene ontology groups, “defense response,” “complement activity,” and “stress response,” were significantly over-represented in the differentially expressed genes in the degenerative retina. The classical complement pathway was found to be upregulated in the *rd1* mouse [51]. The complement pathway has also been implicated in the development of glaucoma and in a model of ocular hypertension [52,53].

Immune-related factors and pathways may thus be important in the process of photoreceptor cell death induced by hyperoxic stress. The present data increase previous understanding of this involvement, but are arguably still inadequate to determine whether immune misregulation causes or results from cell death induced by hyperoxia.

### Retinal disease relevant genes

Seven genes listed in Table 2 were identified as associated with ophthalmic diseases and three of them correspond to known retinal disease loci listed on the RetNet website (*C3,* versican *[Vcan],* and *Nr2e3*). Previous studies reported a significant association between a polymorphism in the *C3* gene and susceptibility to AMD [54,55]. Wagner syndrome can be caused by a mutation in the human *VCAN* gene, which encodes versican, a proteoglycan present in the vitreous body [56]. The syndrome features peculiar lesions of the vitreous and retina, retinal detachment, and progressive chorioretinal dystrophy [56,57]. Versican is widely distributed in the extracellular matrix of a variety of tissues and plays an important role in reducing oxidant injury by enhancing cell-matrix interaction [58]. Mutations in the human *NR2E3* gene, which encodes a retina-specific nuclear receptor, lead to enhanced S-cone syndrome and retinitis pigmentosa [59].

### Antioxidant genes

Metallothioneins (MTs) are a class of low molecular weight, cysteine-rich proteins that bind with copper and zinc. The metallothionein family is composed of four separately encoded isoforms, known as MT-I, -II, -III, and -IV [60]. They serve as important regulators of metal homeostasis and provide cytoprotection in tissues subjected to oxidative stress as a result of their free radical scavenging ability [61]. Increased expression of MT has been reported in various induced models of retinal degeneration, including light [40], hyperoxia [14], and hypoxia [62]. Metallothioneins have been shown to protect RPE cells against toxic levels of cadmium, heme- and iron-induced oxidation, and UV light-induced apoptosis [63]. As endogenous antioxidants, they also protect ganglion cells from oxidative stress caused by the glutamate analog N-methyl-D-aspartic acid (NMDA) [64]. In this study, expression of *Mt1* and *Mt2* is more elevated in the inferior retina that has more tissue damage. The prominence of antioxidants in the retina’s response to hyperoxia indicates the importance of free radicals in the damage caused [14]. Conversely, the vulnerability of the inferior retina does not seem to be due to lack of antioxidants in that region. However, the upregulation of Mt1 and Mt2 are not sufficient for protecting the inferior retina against hyperoxia-induced cell death. This is consistent with previous findings that Mt1 and Mt2 alone are insufficient for protecting photoreceptors against hyperbaric oxygen-induced cell death [16].
